# Disruption of *Nrf2*, a Key Inducer of Antioxidant Defenses, Attenuates ApoE-Mediated Atherosclerosis in Mice

**DOI:** 10.1371/journal.pone.0003791

**Published:** 2008-11-21

**Authors:** Thomas E. Sussan, Jonathan Jun, Rajesh Thimmulappa, Djahida Bedja, Maria Antero, Kathleen L. Gabrielson, Vsevolod Y. Polotsky, Shyam Biswal

**Affiliations:** 1 Department of Environmental Health Sciences, Bloomberg School of Public Health, Johns Hopkins University, Baltimore, Maryland, United States of America; 2 Division of Pulmonary and Critical Care Medicine, Department of Medicine, School of Medicine, Johns Hopkins University, Baltimore, Maryland, United States of America; 3 Department of Molecular and Comparative Pathobiology, School of Medicine, Johns Hopkins University, Baltimore, Maryland, United States of America; Monash University, Australia

## Abstract

**Background:**

Oxidative stress and inflammation are two critical factors that drive the formation of plaques in atherosclerosis. Nrf2 is a redox-sensitive transcription factor that upregulates a battery of antioxidative genes and cytoprotective enzymes that constitute the cellular response to oxidative stress. Our previous studies have shown that disruption of *Nrf2* in mice (*Nrf2* ^−/−^) causes increased susceptibility to pulmonary emphysema, asthma and sepsis due to increased oxidative stress and inflammation. Here we have tested the hypothesis that disruption of *Nrf2* in mice causes increased atherosclerosis.

**Principal Findings:**

To investigate the role of Nrf2 in the development of atherosclerosis, we crossed *Nrf2* ^−/−^ mice with apoliporotein E-deficient (*ApoE* ^−/−^) mice. *ApoE* ^−/−^ and *ApoE* ^−/−^ *Nrf2* ^−/−^ mice were fed an atherogenic diet for 20 weeks, and plaque area was assessed in the aortas. Surprisingly, *ApoE* ^−/−^ *Nrf2* ^−/−^ mice exhibited significantly smaller plaque area than *ApoE* ^−/−^ controls (11.5% vs 29.5%). This decrease in plaque area observed in *ApoE* ^−/−^ *Nrf2* ^−/−^ mice was associated with a significant decrease in uptake of modified low density lipoproteins (AcLDL) by isolated macrophages from *ApoE* ^−/−^ *Nrf2* ^−/−^ mice. Furthermore, atherosclerotic plaques and isolated macrophages from *ApoE* ^−/−^ *Nrf2* ^−/−^ mice exhibited decreased expression of the scavenger receptor CD36.

**Conclusions:**

Nrf2 is pro-atherogenic in mice, despite its antioxidative function. The net pro-atherogenic effect of Nrf2 may be mediated via positive regulation of CD36. Our data demonstrates that the potential effects of Nrf2-targeted therapies on cardiovascular disease need to be investigated.

## Introduction

Cardiovascular disease is responsible for 40% of all deaths in the US and remains a global public health problem [1]. Atherosclerosis, which is characterized by deposition of fatty plaques in the arterial walls, is a leading cause of cardiovascular disease. Atherosclerotic plaques form as a result of macrophage accumulation in the sub-endothelial space of large arteries [2], [3]. Once in the arterial wall, macrophages absorb LDL cholesterol molecules, causing them to develop into foam cells. These cells in turn release chemokines and cytokines, which further enhance inflammatory cell recruitment and perpetuate plaque formation.

Numerous reports indicate that oxidative stress and chronic inflammation are key contributors to the pathogenesis of atherosclerosis. Oxidative stress has several effects on vascular cells, including direct damage of cell membranes [4], and impaired nitric oxide-dependent endothelial-dependent vasomotion [5]. Oxidative stress also causes the oxidation of LDL cholesterol, which enhances LDL uptake by macrophages. Native LDL is not readily absorbed by macrophages. However, oxidized LDL (oxLDL) is rapidly endocytosed by macrophages via scavenger receptors, such as CD36 and scavenger receptor A (SR-A), which localize to the surface of macrophages [2]. oxLDL also has multiple pro-inflammatory properties, including promotion of monocyte adhesion to the endothelial cell wall and increased expression of pro-inflammatory chemokines [6].

Despite ample evidence that oxidative stress plays a key role in atherogenesis, attempts to inhibit oxidative stress have been met with mixed success. In animal models, probucol, vitamin E, butylated hydroxytoluene, and N,N′-diphenylphenylenediamine have been shown to reduce the rate of progression of atherosclerosis [7]–[10]. However, clinical trials of antioxidants in atherosclerosis have not shown any benefits in human subjects [11], [12]. One explanation for this failure is that existing antioxidants are directed against free radicals or single-electron oxidants (superoxide, peroxynitrate), whereas non-radical oxidants, e.g. hydrogen peroxide, remain largely intact [13]. Indeed, deletions of *p47^phox^* and *gp91^phox^* subunits of NADPH oxidase inhibited superoxide generation in the aorta of *ApoE* ^−/−^ mice, but the effects on atherosclerosis were equivocal [14]–[16]; over-expression of Cu/Zn superoxide dismutase (SOD), which converts superoxide to hydrogen peroxide, did not attenuate atherosclerosis in *ApoE* ^−/−^ mice [17]. In contrast, over-expression of catalase, an enzyme decomposing hydrogen peroxide, or both catalase and SOD markedly inhibited the progression of atherosclerotic lesions [17]. This demonstrates that novel therapies targeting oxidative stress should be further pursued.

Nuclear factor E2-related factor 2 (Nrf2) is a member of the basic leucine zipper (bZIP) family of transcription factors that share a conserved cap ‘n’ collar domain. Nrf2 functions as a critical mediator of an adaptive response to counteract oxidative stress. In the presence of oxidative stress, Nrf2 translocates to the nucleus, where it binds to the antioxidant response element (ARE), and activates the coordinate expression of a cohort of antioxidative and electrophile detoxification genes [18]. Activation of Nrf2 causes upregulation of multiple antioxidative enzymes, including several proteins that function to increase the levels of reduced glutathione, which is a major intracellular antioxidant. Our previous studies have shown that mice that are deficient in *Nrf2* are characterized by high levels of oxidative stress and an inappropriate inflammatory response following exposure to a variety of stressors including chronic cigarette smoke-mediated emphysema [19], allergic asthma [20], and endotoxin and acute peritonitis induced septic shock [21].

Several studies suggest that Nrf2 may alter susceptibility to atherosclerosis. Treatment of mouse macrophages with oxLDL causes activation of Nrf2 and increased glutathione synthesis [22], indicating that Nrf2 is an important component of the macrophage response to oxLDL. Also, elevated glutathione levels are protective against atherosclerotic plaques in mice [23], and decreased serum glutathione levels correlate with increased risk of coronary heart disease in humans [24]. Furthermore, the Nrf2-dependent gene heme oxygenase 1 (*Ho-1*) has been shown to reduce the chemotaxis of monocytes following exposure to oxLDL [25], indicating that Ho-1 may inhibit monocyte recruitment to the arterial walls. Accordingly, *Ho-1* deficiency is associated with accelerated progression of atherosclerosis in mice [26].

*In vitro* studies that examined the effects of endothelial shear stress showed that anti-atherogenic laminar flow induces Nrf2-dependent gene expression, while pro-atherogenic oscillatory flow inhibits Nrf2 activity [27], [28], demonstrating that Nrf2 activity may be important for maintaining the structural integrity of endothelial cells. Also Nrf2 decreases expression of vascular cell adhesion molecule 1 (*VCAM-1*) in endothelial cells, suggesting that Nrf2 inhibits monocyte adhesion [28]. On the other hand, Nrf2 increases expression of the pro-atherogenic scavenger receptor CD36 [29], [30]. Despite the indirect evidence that this critical transcription factor may be important in the pathogenesis of atherosclerosis, the role of Nrf2 in atherosclerosis has yet to be investigated in vivo.

To address whether Nrf2 is a modifier of atherosclerosis we utilized *ApoE* ^−/−^ mice, an established model of hyperlipidemia and atherosclerosis. We compared plaque formation and progression in *ApoE* ^−/−^ and *ApoE* ^−/−^ *Nrf2* ^−/−^ mice. Despite the antioxidative function of Nrf2, we observed that *ApoE* ^−/−^ *Nrf2* ^−/−^ mice developed fewer plaques than *ApoE* ^−/−^ controls. We also observed that isolated macrophages from *ApoE* ^−/−^ *Nrf2* ^−/−^ mice showed decreased uptake of modified LDL, which was associated with decreased expression of the scavenger receptor CD36. Thus, Nrf2 is pro-atherogenic in mice.

## Results

### Nrf2 Deficient Mice Exhibit Reduced Atherosclerotic Plaques

**Figure 1:**
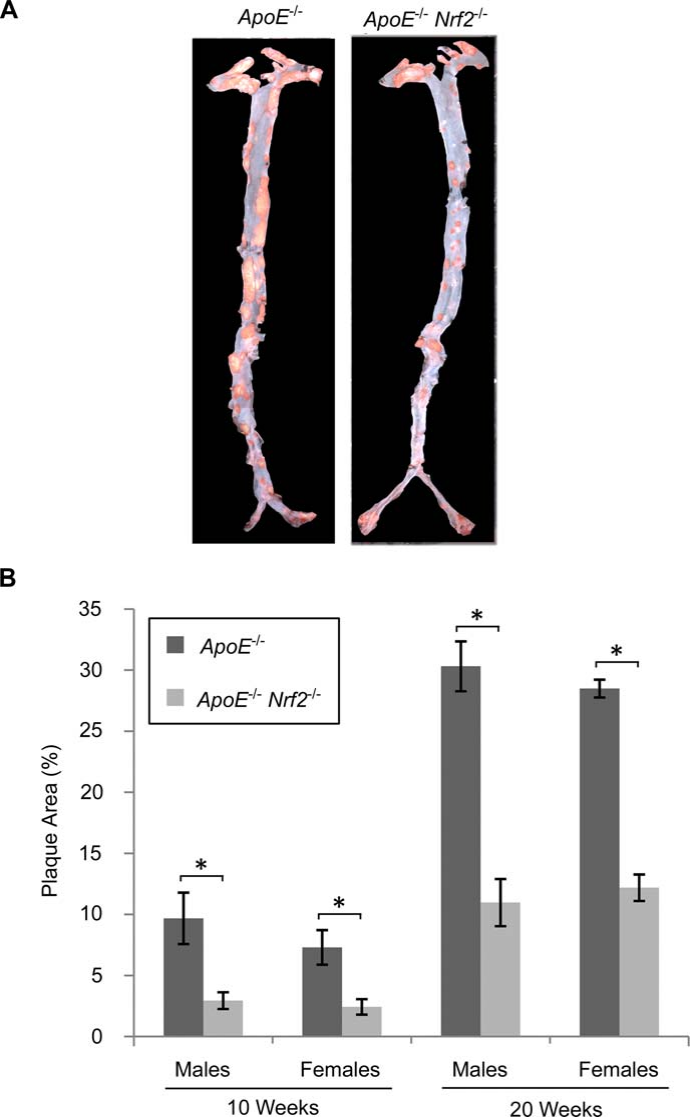
*En face* plaque area of aortas from *ApoE* ^−/−^ and *ApoE* ^−/−^ *Nrf2* ^−/−^ mice. (A) Representative images of aortas from *ApoE* ^−/−^ and *ApoE* ^−/−^ *Nrf2* ^−/−^ mice after 20 weeks on a high fat diet, showing plaques stained with oil red O. (B) Quantification of percent plaque area ±SEM after 10 and 20 weeks on a high fat diet. *p<0.05 by a two-tailed Student's t-test. N = 4–6 per group.

*ApoE* ^−/−^ and *ApoE* ^−/−^ *Nrf2* ^−/−^ mice were fed high fat diet for 10 or 20 weeks, and plaque formation was quantified along the entire aorta in the *en face* preparation. Plaques were located throughout the entire aorta, although the heaviest plaque burden was observed in the aortic arch (Fig. 1). Unexpectedly, *ApoE* ^−/−^ *Nrf2* ^−/−^ mice exhibited a 67% decrease in plaque area after 10 weeks and a 61% decrease after 20 weeks, compared to *ApoE* ^−/−^ mice (p<0.05, Fig. 1A & B). For both genotypes, there was no significant difference between sexes (Fig. 1B).

**Figure 2:**
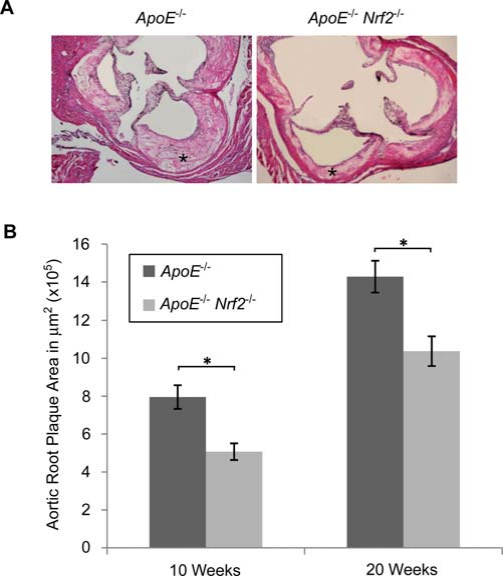
Cross-sectional plaque area at the aortic root. (A) Representative images of aortic cross-sections of *ApoE* ^−/−^ and *ApoE* ^−/−^ *Nrf2* ^−/−^ male mice after 20 weeks on a high fat diet, showing plaques in the arterial walls (asterisks). (B) Quanitfication of plaque area ±SEM after 10 and 20 weeks on a high fat diet. *p<0.05 by a two-tailed Student's t-test. N = 4–6 per group.

At the aortic origin, measurements of cross-sectional plaque area were consistent with the *en face* preparation. Compared to *ApoE* ^−/−^ mice, *Nrf2*-deficient animals exhibited a 36% decrease in plaque size after 10 weeks and a 27% decrease after 20 weeks, which were both statistically significant (Fig 2A & B). As with the *en face* preparation, there was no significant difference between sexes (data not shown). We did not observe significant differences in plaque composition between *ApoE* ^−/−^ and *ApoE* ^−/−^ *Nrf2* ^−/−^ mice in terms of necrotic, fibrotic, or macrophage-rich regions (data not shown).

### Nrf2 Deficient Mice Have Improved Aortic Stiffness

**Figure 3:**
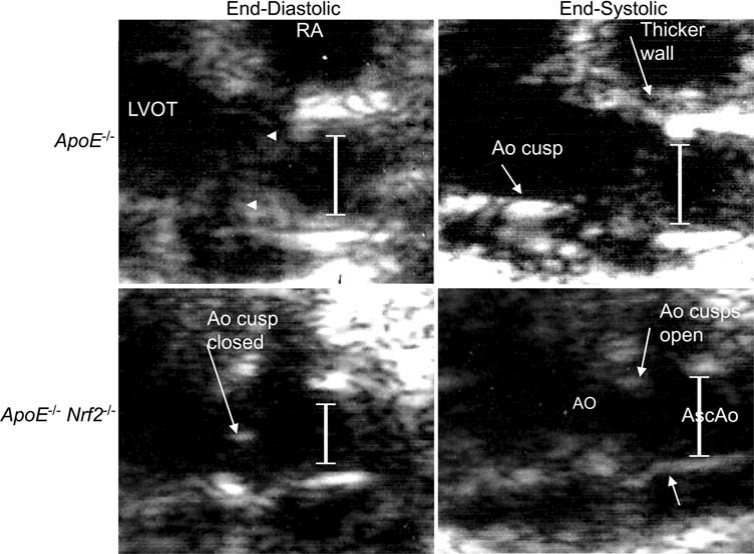
Representative 2 D mode echocardiogram images from *ApoE* ^−/−^ and *ApoE* ^−/−^ *Nrf2* ^−/−^mice following exposure to high fat diet for 20 weeks. Aortic diameters (lines) and plaques (arrowheads) are shown. Aortic root (Ao), ascending aorta (AsAo), right atrium (RA), left ventricle outflow tract (LVOT).

Atherosclerotic plaques cause arteries to become stiffer. We measured aortic diameter at the root, to determine whether the difference in aortic plaques observed in *ApoE* ^−/−^ and *ApoE* ^−/−^ *Nrf2* ^−/−^ mice correlated with altered aortic diameters. Aortic diameter was measured at the aortic sinus during both systole and diastole using two-dimensional echocardiography in mice that were fed a high fat diet for 20 weeks. We observed that *ApoE* ^−/−^ *Nrf2* ^−/−^ mice exhibited significantly decreased aortic end-diastolic diameter, compared to *ApoE* ^−/−^ mice (Table 1 and Fig 3). Also, the percent difference between end-diastolic and end-systolic diameter was significantly increased in *ApoE* ^−/−^ *Nrf2* ^−/−^ mice, suggesting that the aortas from *ApoE* ^−/−^ *Nrf2* ^−/−^ mice were less stiff. This decreased aortic stiffness in *ApoE* ^−/−^ *Nrf2* ^−/−^ mice correlated with the decreased plaque area observed in these mice, demonstrating that atherosclerotic plaques impair normal aortic physiology. As expected, there was no difference between sexes (data not shown).

**Table 1:** End-diastolic and end-systolic diameter of ascending aorta after 20 weeks on a high fat diet.

	End-Diastolic Diam. (mm)	End-Systolic Diam. (mm)	Ratio (ESD-EDD/EDD)
*ApoE* ^−/−^	1.22±0.04*	1.36±0.05	0.11±0.02*
*ApoE* ^−/−^ *Nrf2* ^−/−^	1.07±0.03	1.27±0.02	0.19±0.02

* p<0.05 by a two-tailed Student's t-test.

Values are mean±SEM. Sample size for *ApoE* ^−/−^ and *ApoE* ^−/−^ *Nrf2* ^−/−^ is 9 and 13, respectively.

### Serum Glucose and Cholesterol in ApoE^−/−^ and ApoE^−/−^ Nrf2^−/−^ Mice

To take into account potential differences in the lipid profiles of the two groups, we measured total cholesterol, LDL cholesterol, and triglycerides in fasted *ApoE* ^−/−^ and *ApoE* ^−/−^ *Nrf2* ^−/−^ mice after 20 weeks on a high fat diet. We did not observe any significant change in concentration of serum LDL or total cholesterol between *ApoE* ^−/−^ and *ApoE* ^−/−^ *Nrf2* ^−/−^ mice (Table 2). However, *ApoE* ^−/−^ *Nrf2* ^−/−^ mice exhibited a significant increase in serum triglycerides. Furthermore, we observed that *ApoE* ^−/−^ *Nrf2* ^−/−^ mice exhibited significantly elevated serum glucose concentrations, compared to *ApoE* ^−/−^ mice (Table 2). Since elevated triglycerides and glucose are both associated with promotion of atherosclerosis [1], these measurements do not explain why *ApoE* ^−/−^ *Nrf2* ^−/−^ mice exhibited decreased atherosclerotic plaque formation.

**Table 2:** Serum lipid and glucose levels (total cholesterol, low density lipoprotein, and triglycerides).

	TC (mg/dl)	LDL (mg/dl)	TG (mg/dl)	Glucose (mg/dl)
*ApoE* ^−/−^	739±27	590±20	79±10*	150±5*
*ApoE* ^−/−^ *Nrf2* ^−/−^	706±85	618±40	117±11	201±13

* p<0.05 by a two-tailed Student's t-test.

Values represent mean±SEM. N = 12 per group.

### Nrf2 Deficient Mice Have Elevated Oxidative Stress

**Figure 4:**
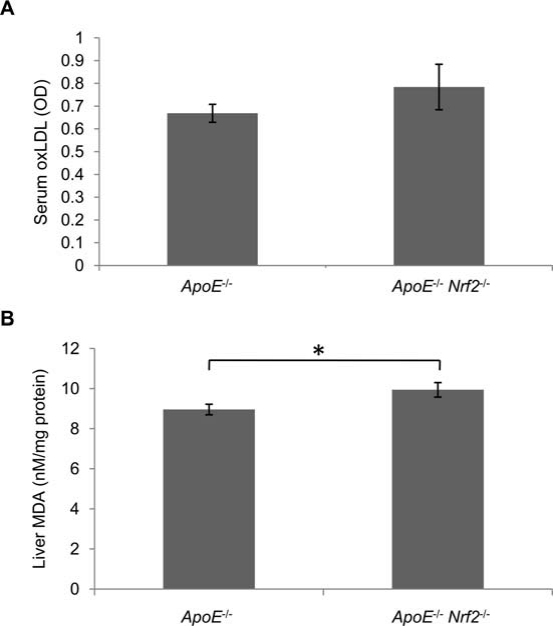
Oxidized LDL and Lipid peroxidation levels in *ApoE* ^−/−^ and *ApoE* ^−/−^ *Nrf2* ^−/−^mice. (A) oxLDL levels were quantified in the serum of mice that were fed a high fat diet for 20 weeks by ELISA. (B) Lipid peroxidation was assessed by quantifying malondialdehyde (MDA) in the liver of *ApoE* ^−/−^ and *ApoE* ^−/−^ *Nrf2* ^−/−^ mice after 20 weeks on a high fat diet. *p<0.05 by a two-tailed Student's t-test. N = 12 per group. Values represent mean±SEM.

Macrophages take up oxLDL more readily than native LDL. Therefore, we measured oxLDL levels in the serum. We did not detect any significant differences in oxLDL levels between *ApoE* ^−/−^ and *ApoE* ^−/−^ *Nrf2* ^−/−^ mice (Fig. 4A). Thus, the decrease in atherosclerotic plaque formation in *ApoE* ^−/−^ *Nrf2* ^−/−^ mice is not due to decreased oxLDL in the serum. We also examined oxidative stress in the liver. The concentration of malondialdehyde (MDA), which is a marker of lipid peroxidation, was elevated significantly in the liver of *ApoE* ^−/−^ *Nrf2* ^−/−^ mice (Fig. 4B). The increased MDA in the liver is consistent with the antioxidative function of Nrf2, but it is contradictory to the decrease in plaque area observed in *ApoE* ^−/−^ *Nrf2* ^−/−^ mice. This suggests that even though Nrf2 reduces oxidative stress, it still promotes atherosclerotic plaque development via a different mechanism.

### ApoE^−/−^ Nrf2^−/−^ Macrophages Exhibited Decreased Modified LDL Uptake

**Figure 5:**
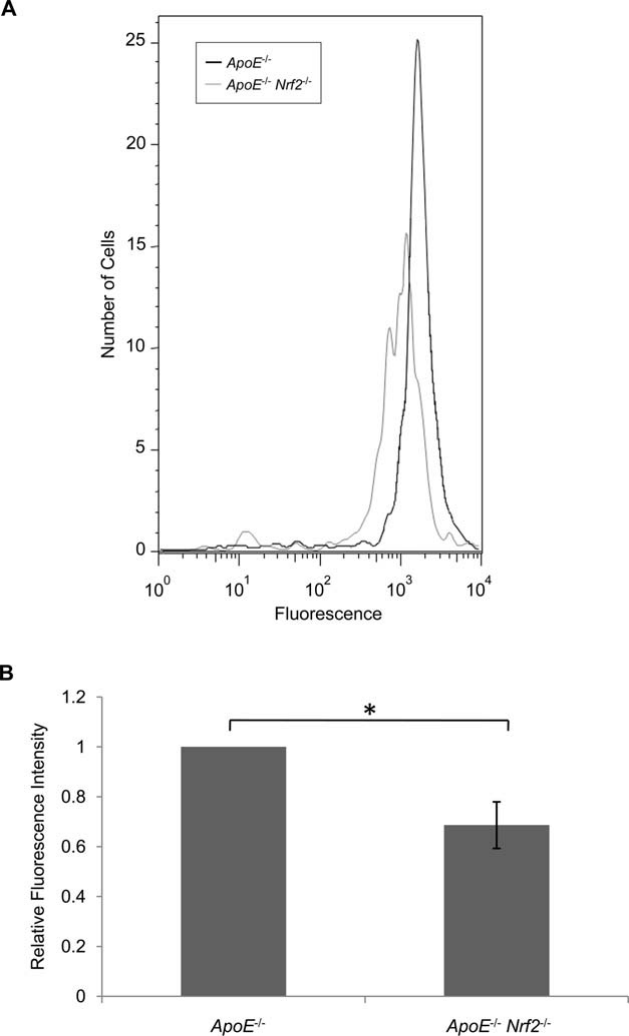
Uptake of AcLDL by bone marrow-derived macrophages from *ApoE* ^−/−^ and *ApoE* ^−/−^ *Nrf2* ^−/−^ mice. Macrophages were incubated with fluorescently-labeled AcLDL for 24 h at 37°C, and AcLDL uptake was assessed by flow cytometry. (A) Representative histograms of fluorescence intensity. (B) Average relative fluorescence intensity of N = 5 mice per group. *p<0.05 by a two-tailed Student's t-test. Values represent mean±SEM.

Macrophages readily absorb modified LDL, via scavenger receptors. Therefore, to determine whether macrophages from *ApoE* ^−/−^ and *ApoE* ^−/−^ *Nrf2* ^−/−^ mice differ in their ability to absorb modified LDL, bone marrow-derived macrophages from *ApoE* ^−/−^ and *ApoE* ^−/−^ *Nrf2* ^−/−^ mice were isolated and incubated with fluorescently-labeled acetylated LDL (AcLDL). AcLDL is a commonly used surrogate for oxLDL. Macrophages from *ApoE* ^−/−^ *Nrf2* ^−/−^ mice exhibited decreased uptake of AcLDL, as assessed by flow cytometry (Fig. 5). Therefore, the decrease in aortic plaques in *ApoE* ^−/−^ *Nrf2* ^−/−^ mice was associated with decreased uptake of AcLDL by macrophages.

### ApoE^−/−^ Nrf2^−/−^ Mice Exhibited Decreased CD36 Expression

**Figure 6:**
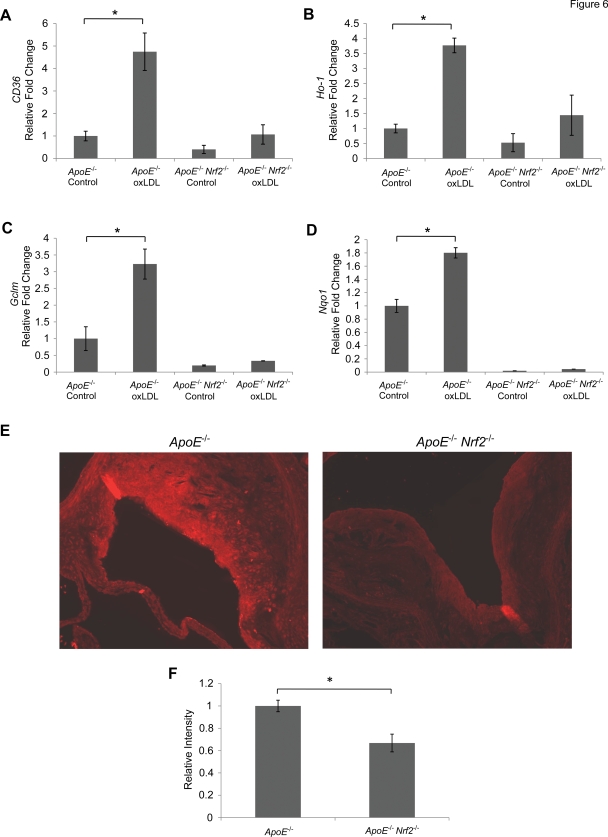
Expression of the scavenger receptor CD36 in macrophages and aortic plaques. Gene expression of *CD36* (A), *Ho-1* (B), *Gclm* (C), and *Nqo1* (D) in bone marrow-derived macrophages, was assessed by quantitative PCR. Macrophages from *ApoE* ^−/−^ and *ApoE* ^−/−^ *Nrf2* ^−/−^ mice were incubated with 50 µg/ml oxLDL for 24 h at 37°C. (E) Representative images of CD36 immunohistochemistry of aorta cross-sections from *ApoE* ^−/−^ and *ApoE* ^−/−^ *Nrf2* ^−/−^ mice that were fed a high fat diet for 20 weeks. (F) Quantification of CD36 staining intensity. *p<0.05 by a two-tailed Student's t-test. N≥3 per group. Values represent mean±SEM.

CD36 is a scavenger receptor that is responsible for recognition of modified LDL, and it plays an important role in plaque development. To determine whether Nrf2 regulates *CD36* expression, bone marrow-derived macrophages were isolated from *ApoE* ^−/−^ and *ApoE* ^−/−^ *Nrf2* ^−/−^ mice. *CD36* expression was determined by quantitative PCR. There was no significant difference in *CD36* expression between *ApoE* ^−/−^ and *ApoE* ^−/−^ *Nrf2* ^−/−^ mice under non-stressed conditions (Fig. 6A). However, treatment of macrophages with oxLDL for 24 h at 37°C caused a significant increase in CD36 expression in *ApoE* ^−/−^, but not *ApoE* ^−/−^ *Nrf2* ^−/−^ mice. Furthermore, *Ho-1*, glutamate-cysteine ligase (*Gclm*), and NAD(P)H:quinone oxidoreductase-1 (*Nqo1*), which are known antioxidative targets of Nrf2, were induced by oxLDL in an Nrf2-dependent manner (Fig. 6B–D). Therefore, despite the function of Nrf2 as a master regulator of antioxidative genes, Nrf2 induces the pro-atherogenic gene *CD36* following exposure to oxLDL. We also determined that gene expression of scavenger receptor A (*Sra)*, *Srb-1*, and low density lipoprotein receptor (*Ldlr*) were not elevated in macrophages from either *ApoE* ^−/−^ or *ApoE* ^−/−^ *Nrf2* ^−/−^ mice in response to oxLDL (data not shown). Furthermore, we did not detect any increases in the pro-inflammatory cytokine *Tnfα* and chemokine *Ccl-2* in response to oxLDL (data not shown).

We also examined *in vivo* levels of CD36 protein within plaques from *ApoE* ^−/−^ and *ApoE* ^−/−^ *Nrf2* ^−/−^ mice that were fed a high fat diet for 20 weeks. CD36 staining was significantly reduced in plaques from *ApoE* ^−/−^ *Nrf2* ^−/−^ mice, compared to *ApoE* ^−/−^ mice (Fig. 6E–F). Thus, Nrf2 increases pro-atherogenic CD36 protein levels in aortic plaques.

## Discussion

Our findings demonstrate that *Nrf2* ^−/−^ mice have a decreased susceptibility to ApoE-mediated atherosclerotic plaque formation. This result was surprising in light of the well-established role of Nrf2 as a critical determinant of the response to counteract oxidative stress. Consistent with this antioxidative role of Nrf2, we demonstrated that *ApoE* ^−/−^ *Nrf2* ^−/−^ mice exhibit significantly elevated lipid peroxidation in the liver, and macrophages from *ApoE* ^−/−^ *Nrf2* ^−/−^ mice failed to induce expression of key Nrf2-regulated antioxidative genes *Ho-1*, *Gclm*, and *Nqo1* in response to oxLDL. Wu *et al.* previously demonstrated that oxidative stress is associated with elevated blood pressure [31]. One limitation of our study is that we did not measure blood pressure in *ApoE* ^−/−^ and *ApoE* ^−/−^ *Nrf2* ^−/−^ mice. However, our data in mice on the C57BL/6 background following 20 weeks on a high fat diet showed that there is no difference in mean blood pressure between wild-type and *Nrf2* ^−/−^ mice (82.2±2.7 and 86.4±3.4 mm Hg, respectively). Hartley *et al.* demonstrated that blood pressure in *ApoE* ^−/−^ mice is not significantly elevated, compared to wild-type mice [32]. Thus, we do not predict any Nrf2-dependent effects on blood pressure.

In response to oxidative stress, Nrf2 activates a battery of antioxidative genes and cytoprotective enzymes, including several enzymes responsible for the synthesis of glutathione [18]. Nrf2 also induces expression of *Ho-1*, which is a key antioxidant enzyme. Both glutathione and *Ho-1* have been shown to prevent atherosclerosis in mice [23], [26]. However, we demonstrated in this study that the master regulator of these antioxidative proteins promotes the development of atherosclerosis. Therefore, it appears as if Nrf2 has conflicting functions in the development of atherosclerosis. In this study, we confirmed the findings of Ishii *et al.* [29] that macrophages lacking Nrf2 take up significantly less modified LDL. Therefore, the antioxidative function of Nrf2 is superseded by its promotion of modified LDL uptake in macrophages.

Macrophages utilize scavenger receptors, such as CD36 and scavenger receptor A (SR-A), to recognize and endocytose modified LDL. *ApoE* ^−/−^ *CD36* ^−/−^ mice internalize less modified LDL and have a substantial decrease in atherosclerotic lesion size, compared to *ApoE* ^−/−^ controls [33]. Our data shows that oxLDL induced *CD36* expression in macrophages from *ApoE* ^−/−^, but not *ApoE* ^−/−^ *Nrf2* ^−/−^ mice. In addition, oxLDL did not induce *Sra*, *Srb-1*, or *Ldlr* expression in *ApoE* ^−/−^ mice, consistent with previous studies that demonstrate the specific role of Nrf2 in the expression of *CD36*, but not of other scavenger receptors [29], [30]. Furthermore, *CD36* has been shown to contain an ARE in its promoter, which is recognized by Nrf2 [30]. We also showed that *ApoE* ^−/−^ *Nrf2* ^−/−^ mice exhibited reduced levels of CD36 protein in the atherosclerotic plaques. Taken together, our data suggest that Nrf2-mediated CD36 expression is a major pathway by which modified LDL becomes incorporated into atheroma. In the absence of Nrf2, even in the face of greater oxidative stress, atherosclerosis is markedly reduced.

We did not observe any significant alteration in the serum levels of total cholesterol or LDL after 20 weeks on a high fat diet, which demonstrates that Nrf2 does not have a net effect on circulating cholesterol. However, we did observe that *ApoE* ^−/−^ *Nrf2* ^−/−^ mice have significantly elevated triglyceride levels. This is consistent with previous studies demonstrating that *CD36*-deficient mice have elevated plasma triglyceride levels [34], [35], further supporting the role of CD36 in *ApoE* ^−/−^ *Nrf2* ^−/−^ mice. Mechanisms of the concomitant increase in blood glucose in Nrf2 deficient mice are less clear, although it may be attributed to dyslipidemia-induced insulin resistance. Our data suggests that *CD36* deficiency in *Nrf2*-deficient mice is a powerful antiatherogenic factor, which can override multiple pro-atherogenic conditions, which were present in the *ApoE* ^−/−^ *Nrf2* ^−/−^ mice, including oxidative stress, hypertriglyceridemia and hyperglycemia. Nevertheless, Nrf2 is a transcription factor that regulates numerous genes, and it is possible that Nrf2 deficiency alters development of atherosclerosis via more than one mechanism.

In conclusion, our data clearly demonstrate that *Nrf2* ^−/−^ mice are less susceptible to ApoE-mediated plaque formation, and this is associated with decreased CD36 expression and decreased LDL uptake by isolated macrophages. Activation of Nrf2 has been proposed as a potential therapeutic approach for a variety of diseases. Considering the profound effect of Nrf2-dependent transcriptional induction of CD36 in macrophages and the resulting impact on atherosclerosis, it is important to consider the potential pro-atherosclerotic effects of Nrf2 when designing Nrf2-targeted therapies.

## Materials and Methods

### Animals and Treatments

*ApoE* ^−/−^ mice maintained on a C57BL/6J background were obtained from Jackson Laboratories (Bar Harbor, ME), and were crossed with C57BL/6J *Nrf2* ^−/−^ mice to generate *ApoE* ^−/−^ *Nrf2* ^+/+^ and *ApoE* ^−/−^ *Nrf2* ^−/−^ mice. Mice were housed under controlled conditions for temperature and humidity, using a 12 h light/dark cycle. Eight week old mice were fed an atherogenic diet (TD.94059) from Harlan Teklad (Madison, WI) for 10 or 20 weeks. For all experiments, mice were age- and sex-matched. When appropriate, data for both sexes was combined. Mice were fasted for 6 h prior to analyses. All experimental protocols were performed in accordance with the standards established by the US Animal Welfare Acts, as set forth in NIH guidelines and in the Policy and Procedures Manual of the Johns Hopkins University Animal Care and Use Committee.

### Measurement of Plaques

For *en face* analysis, aortas were analyzed as described [36]. Briefly, aortas were removed, fixed in 10% formalin, cut longitudinally, stained with oil red O, and photographed. Percent plaque area was measured using Image Pro Express software (MediaCybernetics, Silver Spring, MD). For cross-section analysis of plaque formation, aortas were fixed in 10% formalin and embedded in paraffin. Sections from the aortic root were stained with hematoxylin and eosin, and images were captured with an Olympus BX41 microscope. Area calculations were performed using Image-J software (NIH, Bethesda, MD, USA). Fibrotic areas were estimated based on percent area of intimal plaque positive for Masson's trichrome stain (Richard-Allan Scientific, Kalamazoo, MI). Macrophage-rich areas were identified with Mac3 (BD Pharmingen) immunostaining. 4–8 sections from each animal were used for calculations.

### Aortic Stiffness

Aortic diameter was measured in the ascending aorta of conscious mice via two-dimensional echocardiography, using a Sequoia Acuson C256 (Siemens Medical Solutions USA, Inc., Malvern, PA) ultrasound machine, equipped with a 15 MHz linear transducer. The end-diastolic and end-systolic diameters were determined, and stiffness was estimated as the fractional change in diameter.

### Serum Lipid and Glucose Content

Mice were fasted for 6 h prior to blood withdrawal. Total chosterol, LDL-cholesterol, and triglycerides were measured with kits from Wako Diagnostics, Inc. (Richmond, VA) in 96-well micro-titration plates. For total and LDL cholesterol fractions, serum samples were diluted 6-fold to allow values to fall within the standard curve. Glucose was measured in serum using the ACE glucose reagent (Alfa Wassermann, West Caldwell, NJ) and analyzed using the ACE chemistry analyzer.

### Serum oxLDL and Lipid Peroxidation Measurements

MDA-modified oxLDL was measured using the oxidized LDL ELISA kit (ALPCO Diagnostics, Salem, NH), as described by the manufacturer. For lipid peroxidation measurements, liver tissues were isolated and homogenized in 10 µL/mg ice-cold PBS containing 5 mM butylated hydroxytoluene to inhibit ex vivo oxidation. MDA was assessed by a thiobarbituric acid (TBARS) assay kit (Zeptometrix, Buffalo, NY). Protein concentration was determined by Bradford assay and the value of MDA was normalized to mg protein used in the assay.

### In vitro AcLDL Uptake Assay

Bone marrow-derived macrophages were isolated and cultured. Macrophages were incubated with Alexa 488-conjugated AcLDL (Invitrogen, Carlsbad, CA) in RPMI 1640 medium containing 10% FBS and Pen/Strep for 24 h at 37°C in 5% CO_2_. Cells were washed, harvested, and analyzed using a FACScan flow cytometer (BD Biosciences, San Jose, CA).

### Gene Expression

Bone marrow-derived macrophages were incubated with 50 µg/ml oxLDL (Intracel, Frederick, MD) for 24 h at 37°C. Total RNA was isolated, using the RNeasy mini kit (Qiagen, Valencia, CA), and cDNA was generated using Multiscribe reverse transcriptase (Applied Biosystems, Foster City, CA). Gene expression was measured using assays on demand probe sets (Applied Biosystems), and reactions were analyzed using the ABI 7000 Taqman system. β-actin was used for normalization.

### Immunohistochemistry

Paraffin-embedded cross-sections of the aortic root were stained with a phycoerythrin (PE)-conjugated mouse monoclonal CD36 (ME542) antibody (Santa Cruz Biotechnology, Inc., Santa Cruz, CA). Images were captured on an Olympus BX60 microscope, equipped with a QImaging Retiga EXi camera, and staining intensity was measured using Image-J software.
